# High Pressure-Based Synthesis of Nanoporous Metal–Organic Framework ZIF-93 Giving Rise to a Phase for Proton Conduction

**DOI:** 10.1021/acsanm.5c03130

**Published:** 2025-10-18

**Authors:** Marta Pérez-Miana, Roberto Fernández de Luis, Arkaitz Fidalgo-Marijuan, Junyan Li, Álvaro Mayoral, Joaquín Coronas

**Affiliations:** † Instituto de Nanociencia y Materiales de Aragón (INMA), CSIC-Universidad de Zaragoza, Zaragoza 50018, Spain; ‡ Chemical and Environmental Engineering Department, Universidad de Zaragoza, Zaragoza 50018, Spain; § Basque Center for Materials, Applications and Nanostructures (BCMaterials), UPV/EHU, Leioa 48940, Spain; ∥ Department of Organic and Inorganic Chemistry, University of the Basque Country UPV/EHU, Leioa 48940, Spain; ⊥ Centre for High-resolution Electron Microscopy (ChEM), School of Physical Science and Technology, ShanghaiTech University, Shanghai 201210, China; # State Key Laboratory of Inorganic Synthesis and Preparative Chemistry, College of Chemistry, Jilin University, Changchun 130012, China

**Keywords:** metal−organic framework, zeolitic imidazolate framework, ZIF-93, high-pressure synthesis, solventless synthesis

## Abstract

This study aims to develop a green, solvent-free synthesis of ZIF-93 (ZIF stands for zeolitic imidazolate framework) and to explore the formation of different phases. We report the solvent-free synthesis of a previously unreported nanoporous ZIF phase, ZIF-93_HP (HP referring to “high-pressure”), from zinc oxide using a dual high-pressure (150 MPa) and thermal (110 °C) method. The influence of key synthesis parameters, such as the amount of NH_4_NO_3_ promotor and reaction steps, was systematically investigated to maximize the conversion of ZnO into the intermediate ZIF-93_HP, while, in parallel, preventing its further conversion into nanoporous ZIF-93 phase. The material was extensively characterized by X-ray diffraction, thermogravimetry, electron microscopy and N_2_ and CO_2_ adsorption, which revealed insights into the structure, morphology and nanoporosity of ZIF-93_HP. ZIF-93_HP, with empirical formula of Zn­(C_5_N_2_OH_5_)_2_·1.2­(NH_4_NO_3_)·(H_2_O), is related to the previously reported ZIF-93 (Zn­(C_5_N_2_OH_5_)_2_). Water washing of this phase led to the transformation into ZIF-93 and a significant increase in the BET specific surface area (from 4 to 181 m^2^/g). In addition, the presence of NH_4_^+^ and NO_3_^–^ ions into its structure makes ZIF-93_HP proton conductor at room temperature and moisture conditions (3.76 × 10^–3^ S/cm), a property that decreases with increasing temperature due to dehydration. The discovery of ZIF-93_HP highlights the potential of the high-pressure, solvent-free synthesis as a powerful tool for the exploration of different ZIFs and reticular materials that are inaccessible through traditional solvothermal methods. As crystallization under solvent-free conditions is often influenced by nonthermodynamic equilibrium, this approach holds a great potential for expanding the material landscape by enabling the discovery of different phases and structures with unique properties, such as the promising proton conductivity demonstrated here.

## Graphical abstract

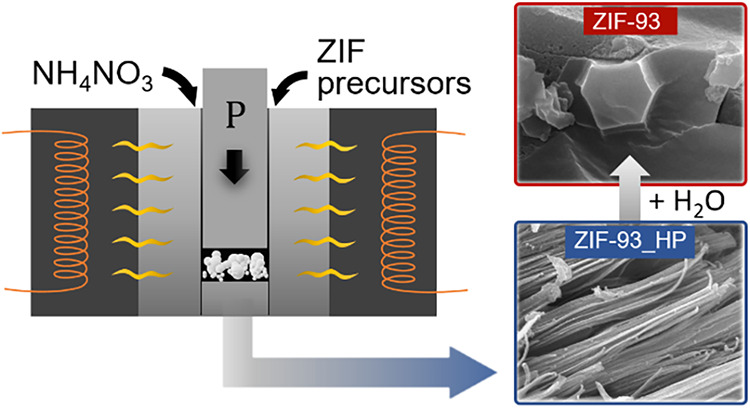

## 1 Introduction

Metal–organic frameworks (MOFs) are crystalline porous materials consisting of an inorganic component (metal ion or metal-oxo cluster) connected with organic ligands through coordination bonds.^1^ Their high surface areas, permanent porosity,^2^ tunable pore environments and chemical versatility^3,4^ make MOFs ideal candidates for a variety of applications, including catalysis,^5^ gas storage,^2,6,7^ membrane molecular separation,^8^ drug delivery^9^ and encapsulation,^10^ just to mention some of the most explored.

Our focus in this study is on a subfamily of MOFs, the zeolitic imidazolate frameworks or ZIFs. They combine zeolite-like topologies with the chemical diversity of imidazolate-type linkers, exhibiting a tetrahedral coordination geometry.^11,12^ ZIF-93 and ZIF-94, which are among the most studied ones, present RHO and SOD-type structures, respectively. They are both composed of Zn ions coordinated with nitrogen atoms from the hydrophilic ligand 4-methyl-5-imidazolcarboxaldehyde (mImca).^13^ Thanks to the presence of polar aldehyde groups, which enhance dipole–quadrupole interactions with CO_2_,^14^ as well as a pore aperture (∼0.36 nm)^15^ close to the molecular sieving threshold, ZIF-93 is especially relevant for gas separation. These combined features provide high affinity and selectivity toward CO_2_ (0.33 nm of kinetic diameter, d_k_) while discriminating against larger molecules such as CH_4_ (d_k_ 0.38 nm), which are well-established principles supported by extensive literature.^13,16–18^ As a result, ZIF-93 has emerged as a promising nanoporous candidate for applications in gas capture and separation, particularly as a filler in mixed-matrix membranes (MMMs) for CO_2_ separation.^19^

Traditionally, most of the ZIFs are synthesized through hydro or solvothermal processes. In detail, one of the original syntheses of ZIF-93 was conducted using zinc acetate in dimethylformamide (DMF).^13^ Later on, several studies moved from harmful and environmentally concerning solvents as DMF to eco-friendly counterparts as water or ethanol. Just to mention an illustrating example, Liu et al.^20^ adopted a hydrothermal method to fabricate ZIF-93 nanocrystals. However, like many other solvothermal synthesis routes, this approach required a high excess of ligand and relied on the use of nongreen methanol for the purification process. After this study, Ramos-Fernandez et al.^21^ optimized the hydrothermal route by using a stoichiometric metal–ligand mixture including NH_4_OH to fasten the imidazole deprotonation and subsequent ZIF-93 precipitation. Even though, the synthesis reaction took more than 18 h. Another alternative synthesis, which employed water and sodium formate as an additive, was reported for the synthesis of this MOF by microfluidics.^22^

Yet, MOF synthesis in aqueous conditions presents several challenges. These include restricted stability of the final product, a tendency toward small, often defective, crystals and side reactions leading to undesired phases.^23,24^ These limitations are primarily originated from thermodynamic factors: the high solvation energy and strong hydrogen-bonding capacity of water molecules can compete with the coordination bonds that drive MOF assembly. This may result in the destabilization of the desired framework and the promotion of alternative crystallization pathways.^25^ In contrast, solvent-free synthesis, particularly under high pressure, introduces dominant thermomechanical effects where mechanical energy and pressure directly influence reaction kinetics and phase selection. These nonequilibrium conditions can access metastable phases that may have unique properties and are inaccessible in solution. Moreover, the elevated specific and latent heats of water can lead to an increased energy consumption during the vaporization process. Additionally, the poor solubility of most organic linkers makes adapting classic synthesis routes of MOFs to water challenging.^26^

These drawbacks have conducted some studies to avoid the use of solvents in the synthesis of some ZIFs. Mechanosynthesis^27^ or high-pressure synthesis^28^ have already been implemented in the synthesis of ZIF-8. These methods do not only eliminate the need of solvents but drastically reduce synthesis times from hours or even days to minutes. This work employs a sustainable, solvent-free synthesis route involving high pressure and temperature, a method we have previously successfully applied to synthesize other ZIFs, such as ZIF-8 and ZIF-L.^28^ Our primary objective was to extrapolate these proven conditions to discover and isolate novel ZIF phases that are inaccessible through conventional methods. In consequence, this high pressure strategy was specifically applied to the synthesis of ZIF-93. We investigated the influence of a promotor (NH_4_NO_3_) and different synthesis steps, parameters critical to the yield and characteristics of the final product, but which were not previously explored for this system. As a result, we successfully isolated a novel phase distinct from ZIF-93 and ZIF-94, named as ZIF-93_HP (“HP” standing for high-pressure). We characterized this new phase and studied its functional properties, such as proton conductivity, to demonstrate the potential of this synthetic strategy for expanding the landscape of reticular materials.

## 2 Experimental Section

### 2.1 Synthesis with Hydraulic Press

The synthesis of ZIF-8 and ZIF-L by means of hydraulic press and high temperature was adapted to ZIF-93 as a starting procedure.^28^ A stoichiometric molar ratio of ligand and metal (2:1) was employed to tentatively obtain a ZIF material with an empirical formula of Zn­(mImca)_2_, mImca being ligand 4-methyl-5-imidazolecarboxaldehyde. This ratio is standard for ZIFs, as it balances the charge of the Zn^2+^ node with two deprotonated imidazolate linkers to form a neutral, extended framework. The synthesis was performed in a hydraulic press (YLJ 15T, MTI corporation) provided with a thermal jacket (500 W), which wraps the cylinder where the reaction takes place (Figure S1). In this study, the temperature (110 °C) and pressure (150 MPa) were fixed at the values reported for the optimized synthesis of ZIF-8. Thus, the effect of the promotor addition, NH_4_NO_3_, was analyzed starting from the amount that yielded the highest efficiencies in ZIF-8 and ZIF-L syntheses, 2.4 and 6.4 wt %, respectively. A comparison of the present synthesis method with three literature procedures for ZIF-93 is provided in Table S1. Figure 1 represents a scheme of the synthesis procedure of ZIF-93_HP in the hydraulic press, followed by the transformation into ZIF-93 when washing with water (as it will be later discussed).

**Figure 1:**
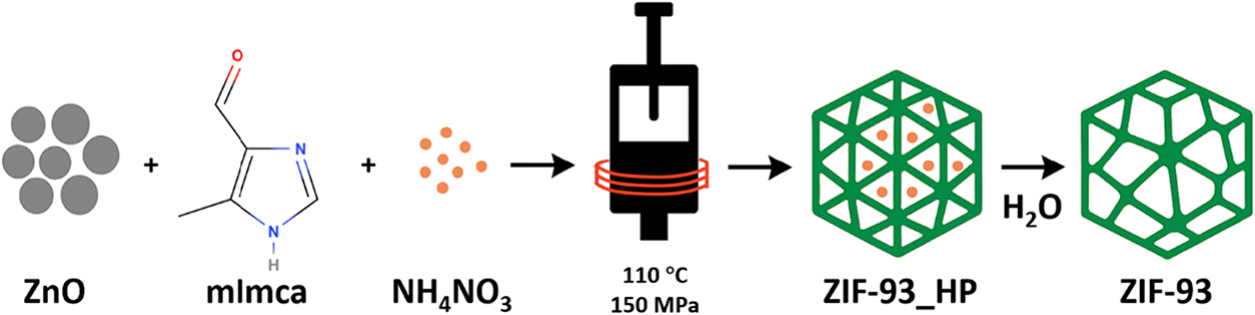
Scheme of the synthesis procedure of ZIF-93_HP and the subsequent transformation into ZIF-93 when washing with water.

### 2.2 Characterization

Powder X-ray diffraction (PXRD) analysis was conducted using a PANalytical Emphyrean-Multipurpose instrument at room temperature conditions. This instrument is equipped with a copper anode and a graphite monochromator, enabling the selection of a monochromatic Cu Kα_1_ radiation with a wavelength of 1.5418 Å. Data were collected within the 2θ range of 2.5–40° at a scanning rate of 0.03°/s.

The PXRD data was first analyzed by a full-profile patching analysis without structural model starting from the cell parameters and the space group determined by transmission electron diffraction (ED) experiments. We skipped the high 2θ – data in the analysis to discard the remnant diffraction maxima arising from ZnO. The fitting of the data enabled extracting the structural factors associated with the nonoverlapped diffraction maxima located at low 2θ angles. Next, SUPERFLIP (a software that applies the charge-flipping algorithm to solve crystal structures from diffraction data)^29^ was employed to calculate the envelope electron density map. By visualizing the electron rich regions within the unit cell, we were able to tentatively assign the positions of the crystallographic independent zinc ions. These were employed to develop an initial rough Rietveld analysis. The attempts to locate and model the imidazole linkers, nitrate and ammonium groups were unsuccessful. However, the analysis of the Zn–Zn distances within the preliminary structure was employed to propose a possible arrangement of the crystal framework. While the final model showed a reasonable fit, it is important to consider that only the Zn ions were located in the structure. Therefore, the results should be interpreted qualitatively, as the absence of other components in the model may limit the accuracy of the proposed structure. A more comprehensive analysis, incorporating additional atoms or ligands, would likely refine the understanding of the overall crystal arrangement.

An experiment of X-ray thermodiffraction (XRTD) was carried out on the PANalytical Empyrean diffractometer using the AP HTK-1200 platform. The wavelengths used in the measurements were Cu Kα_1_: 1.540598 Å and Cu Kα_2_: 1.544426 Å (with a Kα_2_/Kα_1_ intensity ratio of 0.50). The XRTD patterns were acquired within the 2θ range of 2.5–40°, from room temperature (25 °C) up to 300 °C. The duration of the full data acquisition at each temperature was 135 min. All the measurements were conducted at atmospheric pressure.

Thermogravimetric analysis (TGA) was performed utilizing the Mettler Toledo equipment TGA/SDTA 851e. The samples were placed in alumina pans (70 μL) and heated up to 700 °C at a 10 °C/min heating rate under a synthetic air atmosphere. TGA data was used to calculate the reaction yield, as detailed in the eq 1. The ZnO yield to ZIF-93 was estimated from the stoichiometric formula, Zn­(mImca)_2_, considering ZnO and mImca in dry basis in pure ZIF-93. In order to discard the unreacted ligand and solvent from the calculations, the weight loss (%) observed at the TGA at 380 °C was normalized to the 100%. Thus, the weight loss observed from 380 °C up to the complete degradation of the sample was ascribed to the calcination of the *mImca* in the sample (eq 1). To calculate the yield of the new phase, named as ZIF-93_HP, it was considered as yield to the stoichiometry of ZIF-93, i.e., excluding the amount of NH_4_NO_3_ and water present in the empirical formula (by normalizing as well the TGA data to the 100% at 380 °C) as it will be explained below. The amounts of ZnO and mImca calculated in 100 g of ZIF-93 are 28.70 and 76.94 g, respectively.

1 $$yield\left(\%\right)=\frac{mImca\;in\;sample\left(\%\right)\cdot \frac{28.70\;g\;ZnO\;in\;ZIF-93}{76.94\;g\;mImca\;in\;ZIF-93}}{\left(100-mImca\;in\;sample\left(\%\right)\;g\;ZnO\;in\;sample\right)}\cdot 100$$

Elemental analysis was measured using a PerkinElmer Series II 2400 CHNS/O Analyzer. It was carried out using the CHNS method without optimizing the oxygen input, which means without providing extra seconds of oxygen flow for enhanced combustion. Weighing was conducted at room temperature with exposure to air until it was encapsulated in tin capsules. The microbalance used is the Provectus 6500, also from PerkinElmer, and it is connected to the analyzer.

Scanning electron microscopy (SEM) was conducted using an Inspect-F microscope (FEI) operating at 10 kV and a working distance of 10 mm. The samples were prepared by coating with Pd while situated on a magnetic strip within a vacuum environment. Transmission electron microscopy (TEM) images were obtained using a Tecnai G2 T20 (FEI) operating at 200 kV.

Three-dimensional electron diffraction (3D-ED) data sets were collected using a continuous tilting method by a modified INSTAMATIC software^30^ with an ASI Cheetah 1800 hybrid pixel detector equipped on a JEOL JEM-F200 transmission electron microscope at 200 kV. Data sets were reconstructed and reciprocal sections were processed to gain the reflection conditions by an ED data-process software.^31^ The camera has 512 × 512 pixels, each with a physical size of 55 μm. Selected area electron diffraction patterns were collected at a calibrated camera length of 443 mm, corresponding to a pixel size of 0.00495 1/Å in each diffraction pattern using a JEOL JEM-F200 transmission electron microscope operating at 200 kV.

Nitrogen adsorption–desorption isotherms were measured with a Micrometrics Tristar 3000 instrument with N_2_ at the temperature of 77 K. The samples were previously degasified under vacuum conditions for 8 h at 200 °C. The Brunauer–Emmett–Teller (BET) method was applied to determine the BET specific surface area (SSA). CO_2_ adsorption isotherms were obtained using a Micromeritics ASAP 2020 at 273 K. Prior to measurements, two successive degasifications were performed at 200 °C with a heating rate of 10 °C/min under vacuum for 8 h.

### 2.3 Proton Conductivity Assessment

For the conductivity measurements, the ZIF-93_HP powder (ca. 120 mg) was pressed at 10 tons for 5 min to form a compact disc of 10 mm diameter and 0.652 mm thick. The temperature was measured by means of a type K thermocouple in contact with the sample and the relative humidity (RH) was controlled using a saturated aqueous solution of K_2_SO_4_ (≈97% RH). The electrical properties were determined for the plane-parallel sample, performing alternating current (AC) complex impedance measurements with a Solartron 1260 Impedance Analyzer. The measured frequency range was 10^–1^–10^6^ Hz, with a 10 mV signal amplitude. The behavior of the material was studied by heating from room temperature to 80 °C. The impedance diagrams were analyzed and fitted by the Zview software. The conductivity values (σ) were calculated using the following expression

2 $$\sigma =\frac{L}{R\cdot A}$$

where *L* (cm) and *A* (cm^2^) are the thickness and surface area of the pellet, respectively, and *R* (ohm) is the resistance of the sample obtained from the intersection of the curve with the real axis in the Nyquist diagram.

## 3 Results and Discussion

In this work, we have extrapolated the solventless high-pressure synthesis at moderate temperature of ZIF-8 and ZIF-L^28^ to ZIF-93. In contrast to grinding-based mechanosynthesis, the main advantage of static pressure and temperature-driven synthesis is its ability to reduce mechanical deformation and prevent shear-induced loss of crystallinity, thereby reducing its impact on particle size and shape, as well as the risk of amorphization.^32^ To synthesize ZIF-93, ZnO was employed as the zinc source due to its reactivity, already demonstrated through the mechanosynthesis of several ZIFs.^27,32,33^ Previous attempts by Paseta et al. to utilize various zinc salts beyond ZnO in the high-pressure method did not yield apparent reactions.^34^ Some of the options were also discarded due to concerns regarding the potential formation of acids (HCl, HNO_3_, HAc, etc.) and their corrosive effects on the mechanical components of the press.

As it will be explained in detail in the following sections, we were able to crystallize a new ZIF phase from this high pressure solventless synthesis (ZIF-93_HP) and later transform it to ZIF-93 upon contact with some polar solvents. To do so, we first optimized the overall yield of the ZnO to ZIF-93_HP reaction by modulating the concentration of the NH_4_NO_3_ promotor. Later, the ZIF-93_HP to ZIF-93 transformation was followed by selecting the proper solvent.

### 3.1 Addition of Promotor NH_4_NO_3_

First, a synthesis without NH_4_NO_3_ was conducted for comparison with reactions using this promotor, assessing its influence and potential yield improvement, as previously reported for the synthesis of ZIF-8 and ZIF-L.^28^ The PXRD pattern in Figure 2a indicates that a residual amount of ZIF-93 (i.e., a yield of 20.8%) crystallized without the addition of any promotor. The PXRD patterns of the pure ZIF-93, synthesized by a conventional protocol and the mImca organic linker are shown in the same figure as references. In the following runs, 2.4 and 6.4 wt % of NH_4_NO_3_ were added to the synthesis media in order to improve the reaction yield. Consistent with previous observations,^28^ the NH_4_NO_3_ addition, and more specifically the acid character of NH_4_^+^ cations, promotes the dissolution of ZnO, following eq 3. The NH_3_ generated during the process deprotonates HmImca (as described in eq 4), which in turn reacts with the ionized Zn (eq 5) to form a ZIF material with a 2:1 metal to linker stoichiometry.

$$\mathrm{ZnO}+2{\mathrm{NH}}_{4}{\mathrm{NO}}_{3}\to {\mathrm{Zn}}^{2+}+2{\mathrm{NH}}_{3}+H_{2}O+2{\mathrm{NO}}_{3}^{-}$$ 3

4 $${\mathrm{NH}}_{3}+\mathrm{HmImca}\to {\mathrm{NH}}_{4}^{+}+{\mathrm{mImca}}^{-}$$

5 $${\mathrm{Zn}}^{2+}+2{\mathrm{mImca}}^{-}\to \mathrm{Zn}{\left(\mathrm{mImca}\right)}_{2}$$

**Figure 2:**
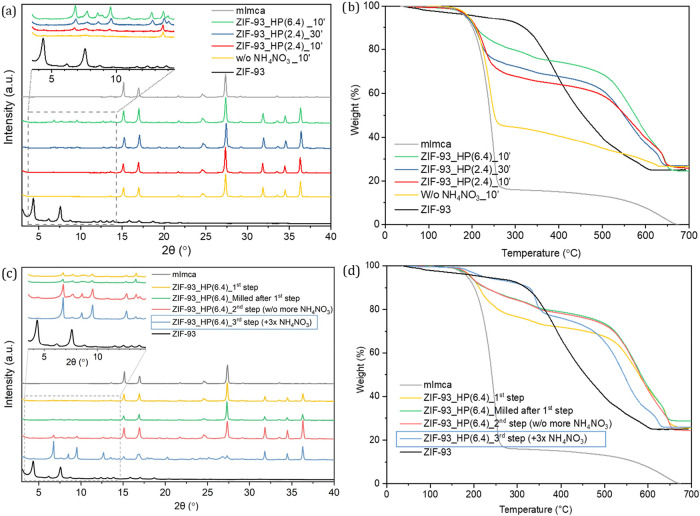
PXRD patterns (a) of samples with different amounts of promotor NH_4_NO_3_ and their corresponding TGA curves (b). PXRD patterns (c) of sample ZIF-93_HP(6.4) and different reaction steps and their corresponding TGA curves (d). ZIF-93 (black) and mImca (gray) were included for comparison.

The application of high pressure and moderate temperature in this solvent-free system provides the necessary activation energy to overcome the kinetic barriers associated with solid-state reactions, primarily facilitating the dissolution of the ZnO precursor and its subsequent reorganization into the crystalline ZIF framework.^35^ While the initial disruption of the solid ZnO precursor is energetically costly, the overall process is achieved by the highly favorable thermodynamics of forming strong Zn–N coordination bonds, a principle that supports the synthesis of all zeolitic imidazolate frameworks, including ZIF-93.

As a first insight, 4 samples were analyzed by PXRD: the sample obtained without NH_4_NO_3_, already discussed, two samples synthesized with 2.4 and 6.4 wt % of NH_4_NO_3_ reacted at 110 °C and 150 MPa during 10 min, and a last one synthesized with 2.4 wt % of promotor at the same conditions but during 30 min. The selected reactions enable evaluating the influence of both the promotor concentration and the reaction time in the system. PXRD patterns of the samples after the reaction (Figure 2a) revealed that instead of the expected ZIF-93 or ZIF-94 (both composed by the same reactants), a new crystalline phase was obtained when adding NH_4_NO_3_. The main diffraction intensities of these compounds appear at 6.8, 7.7, 8.6 and 9.5°. According to TGA (shown in Figure 2b), the new phase presents a thermal stability above 500 °C, with around 20% (varying depending on the sample yield) of unreacted ligand removed at around 200 °C. It is important to note at this point that since water and NH_4_NO_3_ are products of the proposed reactions, they could integrate part of the ZIF-93_HP structure during the formation process of this material, as it will be later discussed. Therefore, we hypothesize that NH_4_NO_3_ primarily functions as a dissolution agent (as explained above) and kinetic modulator. The incorporation of both NH_4_^+^ and NO_3_^–^ ions into the ZIF-93_HP framework (as confirmed by elemental analysis and TGA, later discussed) indicates they have two main functions. They act as structure-directing agents for the nucleation and stabilize the metastable ZIF-93_HP phase during growth and also act kinetically impeding the formation of the thermodynamically favored ZIF-93.

Table 1 collects the reaction yields calculated from TGA data, which revealed an improvement in the conversion from 23.5 to 76.5% correlated with the NH_4_NO_3_ content in the reaction (from 2.4 to 6.4 wt %). For the sample obtained from a 2.4 wt % of promotor, the yield also increases from 23.5 to 57.3% as the reaction time is increased from 10 to 30 min. Samples obtained from 2.4 and 6.4 wt % of promotor and 30 and 10 min of reaction, respectively, were used for further reaction optimization. From now on, they will be referred as ZIF-93_HP(2.4) and ZIF-93_HP(6.4), respectively.

**Table 1:** Reaction Yields to the Formation of ZIF-93 of Samples with Different Amounts of Promotor NH_4_NO_3_

sample	temperature and pressure	NH_4_NO_3_ (wt %)	reaction time (min)	yield (%)
W/o NH_4_NO_3__10’	110 °C, 150 MPa	0	10	**20.8**
2.4 wt % NH_4_NO_3__10’ **(ZIF-93_HP(2.4)_10’)**	110 °C, 150 MPa	2.4	10	**23.5**
2.4 wt % NH_4_NO_3__30’ **(ZIF-93_HP(2.4)_30’)**	110 °C, 150 MPa	2.4	30	**57.3**
6.4 wt % NH_4_NO_3__10’ **(ZIF-93_HP(6.4)_10’)**	110 °C, 150 MPa	6.4	10	**76.5**

Experiments without pressure and 2.4 wt % of NH_4_NO_3_ were also performed during 10 min at 110 °C to check their influence in two different approaches. One of them was carried out just by introducing the solid reactants in press (previously mixed by hand shaking) without using the piston. The same experiment was performed using the minimum pressure, just below the detection threshold of the press manometer. As indicated by the PXRD data and TGA curves (in Figure S2a,b, respectively), compacting reactants already has a positive influence on the reaction, as it improves the contact between the solid precursors, the ZIF-93_HP phase being detected when applying a small pressure in the system.

### 3.2 Cumulative Reaction Steps

Starting from the synthesis conditions for ZIF-93_HP(2.4) and ZIF-93_HP(6.4), the reaction was repeated (up to 3 times) under the same conditions (110 °C, 150 MPa and 30 or 10 min, respectively) after softly milling the pellets obtained in the first reaction step. The samples were analyzed after the milling and high pressure/temperature reaction. PXRD and TGA data in Figure S3a,b reveals that sample ZIF-93_HP(2.4) shows a minimal additional reaction after grinding or pressing. In contrast, the analysis of the PXRD data (Figure 2c) for ZIF-93_HP(6.4) pointed that the reaction slightly evolves after milling, and especially under the following pressure and temperature steps. These facts indicate that NH_4_NO_3_ continues promoting the transformation of unreacted precursors when a second cumulative milling and pressing process is applied to the sample. These results suggest that milling and pressing again may redistribute unreacted precursors improving their contact. The ZIF crystals already synthesized in the first press step may also act as seeds, possibly enhancing the growth of crystals^36^ during the subsequent cumulative milling and hot-pressing steps (even without the further addition of promotor).

As the experimental facts point that the presence of unreacted NH_4_NO_3_ is driving the ZnO to ZIF-93_HP transformation, the cumulative addition of NH_4_NO_3_ during consecutive hot-pressing steps was studied in the following step. Three cumulative reactions with the addition of the same amount of NH_4_NO_3_ in every step were carried out for samples ZIF-93_HP(2.4) (Figure S3) and ZIF-93_HP(6.4) (Figure 2c,d). The promotor was incorporated and homogenized by soft milling before the reaction was initiated by hot-pressing. As revealed by PXRD, the cumulative milling and hot-pressing enhance the crystallization of ZIF-93_HP while reducing the intensity of the diffraction maxima ascribed to the organic linker and ZnO precursors. This improvement of the reactivity was quantified by the analysis of the TGA curves (Figures 2d and S3b). In detail, the reaction yields for ZIF-93_HP(6.4) and ZIF-93_HP(2.4) after 3 steps of soft-milling and hot-pressing with promotor addition increase up to 73.0 and 76.4%, respectively. The increase of yield with time and with promotor concentration highlights the kinetic nature of this transformation. The data suggest that the concentration of NH_4_NO_3_ is the primary factor in enhancing the reaction rate, likely by accelerating the dissolution of the ZnO precursor. At the highest NH_4_NO_3_ loading, the system apparently approaches a saturation limit indicating a possible change in the rate-limiting step from precursor dissolution to another process, such as mass transport within the solid matrix or the intrinsic rates of crystal nucleation and growth.

The reaction yield for ZIF-93_HP(6.4) after 3 steps was found to be 73.0 ± 2.8% (*n* = 3), confirming the good reproducibility of the method. The high consistency of the PXRD patterns between batches (Figure S4) also indicates the reproducibility of the phase formation. The samples obtained after the three cumulative reaction cycles will be named hereafter as ZIF-93_HP_3×(2.4) and ZIF-93_HP_3×(6.4), respectively.

Overall, the experimental results suggest that the conversion of ZnO to ZIF-93_HP under hot-pressing conditions depends on the presence of a sufficient amount of NH_4_NO_3_ in the reaction medium. Meanwhile, soft milling between hot-pressing steps promotes the redistribution of unreacted precursors and enhances the contact between solid reactants. This process is possibly aided by particles formed in the initial pressing step, which may act as seeds for secondary nucleation and crystal growth.

### 3.3 ZIF-93_HP in Contact with Different Solvents

The presence of the unreacted ligand in ZIF-93_HP(2.4) but also in ZIF-93_HP(6.4) was revealed in the weight loss at around 200 °C in TGA (Figures S3b and 2d, respectively), as well as by the observation of the main diffraction maxima of the mImca phase at 27.3° in the PXRD patterns (Figures S3a and 2c, respectively). Thus, the washing process of the samples was studied to eliminate the unreacted components.

Protic and nonprotic and polar and nonpolar solvents were selected to wash the products, covering a wide range of polarity: H_2_O, EtOH, toluene and octanol (OctOH). The dielectric constants (ε) of these solvents (H_2_O, ε = 80.1; EtOH, ε = 24.3; OctOH, ε = 10.0; and toluene, ε = 2.4) provide a quantitative tool for understanding the washing results. After washing, PXRD revealed changes in the crystallinity directly correlated with solvent polarity (Figures 3 and S5). Highly polar H_2_O completely transformed ZIF-93_HP into ZIF-93, mainly due to efficient extraction of the NH_4_^+^ and NO_3_^–^ ions. EtOH, with moderate polarity, led to a partial transformation. In parallel, nonpolar toluene maintained the crystallinity of the parent ZIF-93_HP while reducing the intensity of the diffraction signature associated with the linker. In comparison, the washing process with OctOH, being weakly polar, just slightly removed the unreacted ligand for the final sample without inducing any phase change. The severe reduction in the diffraction intensity after H_2_O washing is consistent with the conversion of ZIF-93_HP into the stable ZIF-93 phase. Figure 3 collects the PXRD patterns corresponding to sample ZIF-93_HP_3×(6.4), while those of sample ZIF-93_HP_3×(2.4) were included in Figure S5.

**Figure 3:**
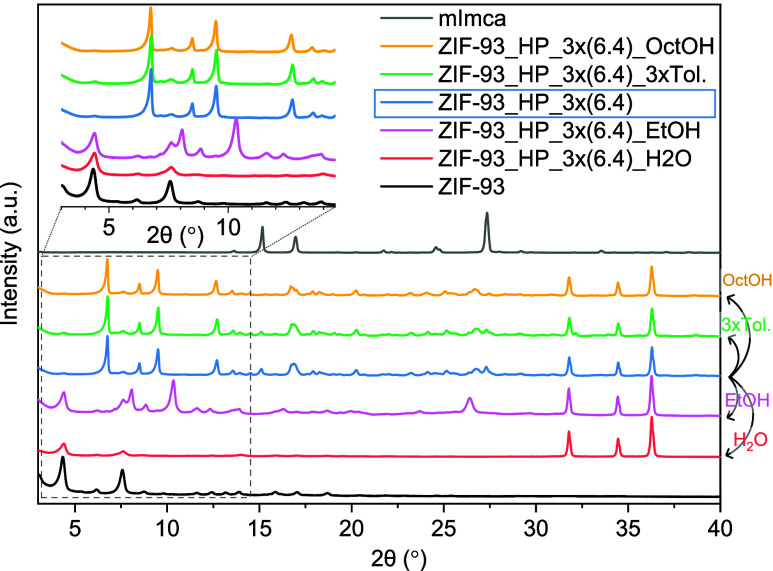
PXRD patterns of sample ZIF-93_HP_3×(6.4) before washing (blue line) and after washing with different solvents.

TGA curves of the samples after the washing protocols are collected in Figure S6a (ZIF-93_HP_3×(6.4)) and Figure S6b (ZIF-93_HP_3×(2.4)). Samples washed with water (red line) revealed an important increase in the final amount of ZnO, which is in line with the conclusions drawn from the PXRD data: partial transformation of ZIF-93_HP phase together with unreacted linker and NH_4_NO_3_ components. Washing with toluene or OctOH did not reveal a phase transition since NH_4_NO_3_ shows minimal solubility in both of these solvents. This allows inferring that the removal of the salt from the structure (achieved with a highly polar solvent) drives the transformation of ZIF-93_HP phase toward ZIF-93. As the sample washed with OctOH showed the most efficient removal of unreacted linker from the sample (without inducing the ZIF-93_HP to ZIF-93 transformation) it was selected for further characterization by electron microscopy and thermodiffraction. The sample was dried at 200 °C for 5 days to ensure the full removal of OctOH, as confirmed by thermogravimetry (Figure S6c).

SEM and TEM analyses were performed to elucidate the shape of ZIF-93_HP before and after its transformation into ZIF-93 during washings. Figure 4a,b,c belongs to parent ZIF-93_HP_3×(6.4) (without washing) at different magnifications. The SEM images reveal a particular rod-like shape in the nanosized-scale with around 50 nm of thickness. After washing with OctOH (Figure 4d,e,f), the morphology remained similar to that of the parent sample. In some areas, besides the rod-like morphology tentatively ascribed to ZIF-93_HP, the distinctive dodecahedral-like morphology of ZIF-93 can be also observed (as shown in Figure 4f). In agreement with the PXRD data (zoomed-up inset in Figure 2c), the presence of ZIF-93 in the ZIF-93_HP_3×(6.4) sample is secondary in comparison to the main ZIF-93_HP component.

**Figure 4:**
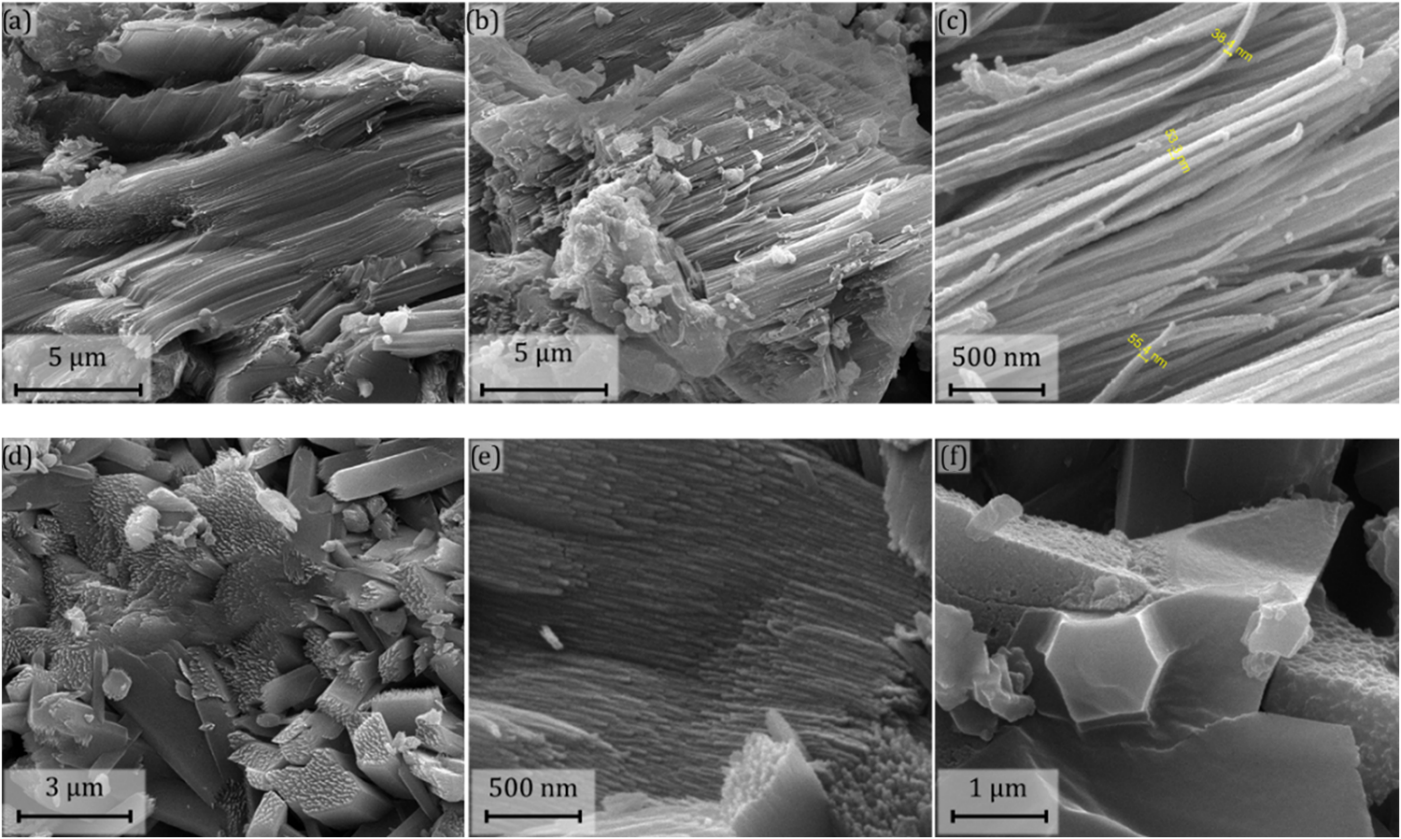
SEM images of sample ZIF-93_HP_3×(6.4) before washing (a, b and c; at ×16.000, ×16.000 and ×120.000 magnifications, respectively) and after washing with octanol (d, e and f; at ×24.000, ×120.000 and ×60.000 magnifications, respectively).

TEM images give a complementary information on the morphology of the samples at the nanometric scale, as observed in Figure 5. First, ZIF-93_HP_3×(6.4) before washing (Figure 5a,b,c) exhibits two different morphologies: (i) a core–shell structure with an unreacted ZnO core covered with a ZIF-93_HP shell. In the same sample, (ii) prismatic crystals, which could be comprised of compacted fibrils observed in the SEM images (i.e., Figure 4d), are as well depicted (Figure 5b). As Figure 5c shows, in the same sample, isolated crystals that may correspond to ZIF-93 are observed, similarly to the ones found by SEM image (Figure 4f). Figure 5d and e illustrate the TEM images of the same sample after washing with OctOH. Given the morphology of the crystals, the ones observed in the Figure 5d are tentatively ascribed to the ZIF-93 phase, while prisms composed by stacked rods in Figure 5e (similar to the SEM ones in Figure 4d) are associated with ZIF-93_HP. The same sample, after washing with water, was characterized by TEM (Figure 5f), showing a spherical nanometric interconnected morphology that could be linked to the partial dissolution of the ZIF-93_HP phase and its transformation into ZIF-93.

**Figure 5:**
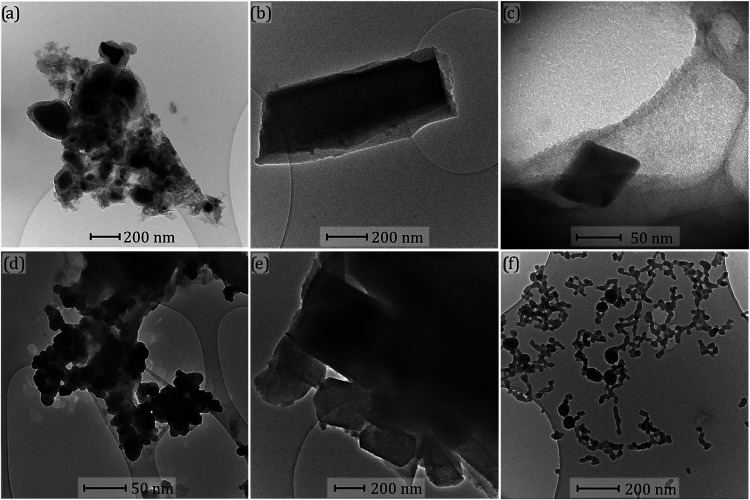
TEM images of sample ZIF-93_HP_3×(6.4) before washing (a, b and c), washed with OctOH (d and e) and washed with water (f).

### 3.4 Structural Analysis of ZIF-93_HP

High-resolution TEM imaging was attempted in sample ZIF-93_HP_3×(6.4) washed with toluene; however, it was found to be highly unstable under the electron beam. We then turn into electron diffraction (ED) as it allows working under lower dose conditions. 3D-ED was performed to extract the cell parameters and the possible space group of ZIF-93_HP. Figure 6 shows the projections along the [100], [010] and [001] directions. After data collection, the space group was determined to be *P*2/*n* with unit cell constants of *a* = 14.82 Å, *b* = 7.69 Å, *c* = 18.45 Å, α = γ = 90.000° and β = 100.889°. Further structural ab initio solution and refinement could not be accomplished due to the low data quality associated with low beam stability. A more accurate structural determination, including definitive space group assignment, would require data from high-resolution techniques (such as synchrotron and single crystal analyses).

**Figure 6:**
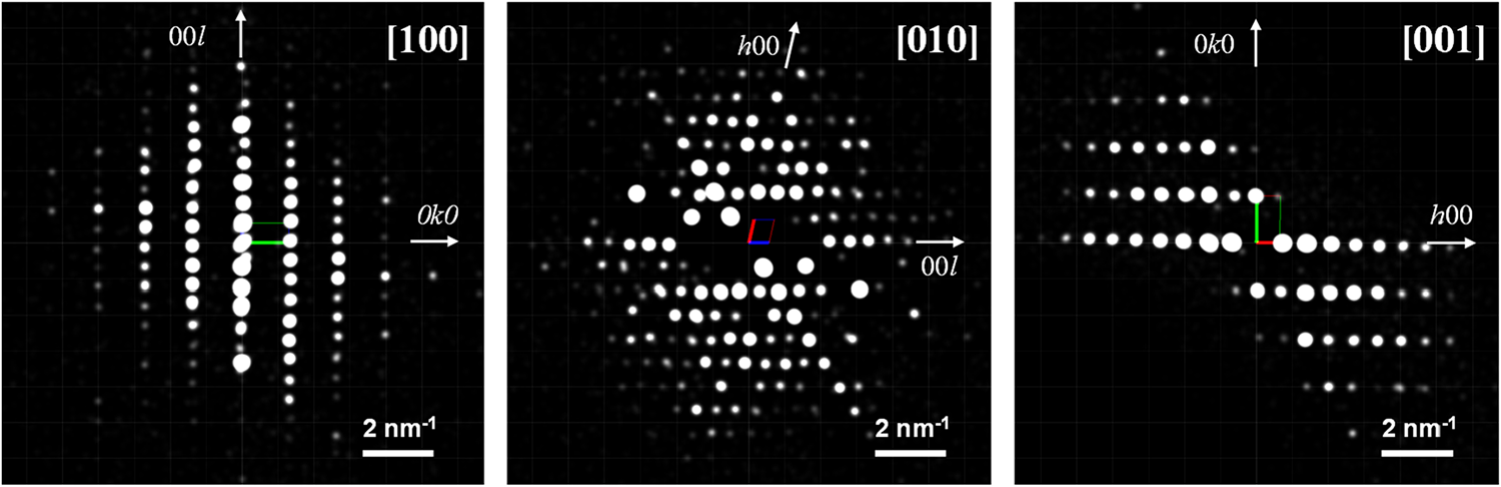
ED patterns obtained from one crystal of sample ZIF-93_HP_3×(6.4) washed with toluene along the main crystallographic orientations and indexed assuming *P*2/*n* symmetry. The ED patterns were reconstructed from the 3D-ED data set.

Even though, the crystallographic information obtained during the process was later applied to face the analysis of the PXRD data. The X-ray diffraction pattern of ZIF-93_HP_3×(6.4) was selected for its fitting, giving the quality of the data associated with the ZIF-93_HP phase in this sample. The PXRD data was first fitted employing a profile pattern analysis without structural model. Regardless of some residual impurities, the positions of the main maxima fit with the cell parameters and the space group obtained from ED. It is important to note that the broadening and the asymmetry of the maxima, likely arising from strain and/or structural disorder of the material, worsen the data refinement. Even though, the profile fitting allowed obtaining the structural factors of the nonoverlapped reflections located at low 2θ (°) region. The structural factors were employed to obtain the envelope density map of the framework.^37^ As shown in Figure 7a–c, the electron density-rich regions of the structure are located in (xy0) and (xy0.5) crystallographic planes. Those are as well connected along the [001] crystallographic direction via electron-density rich like- pillars.

**Figure 7:**
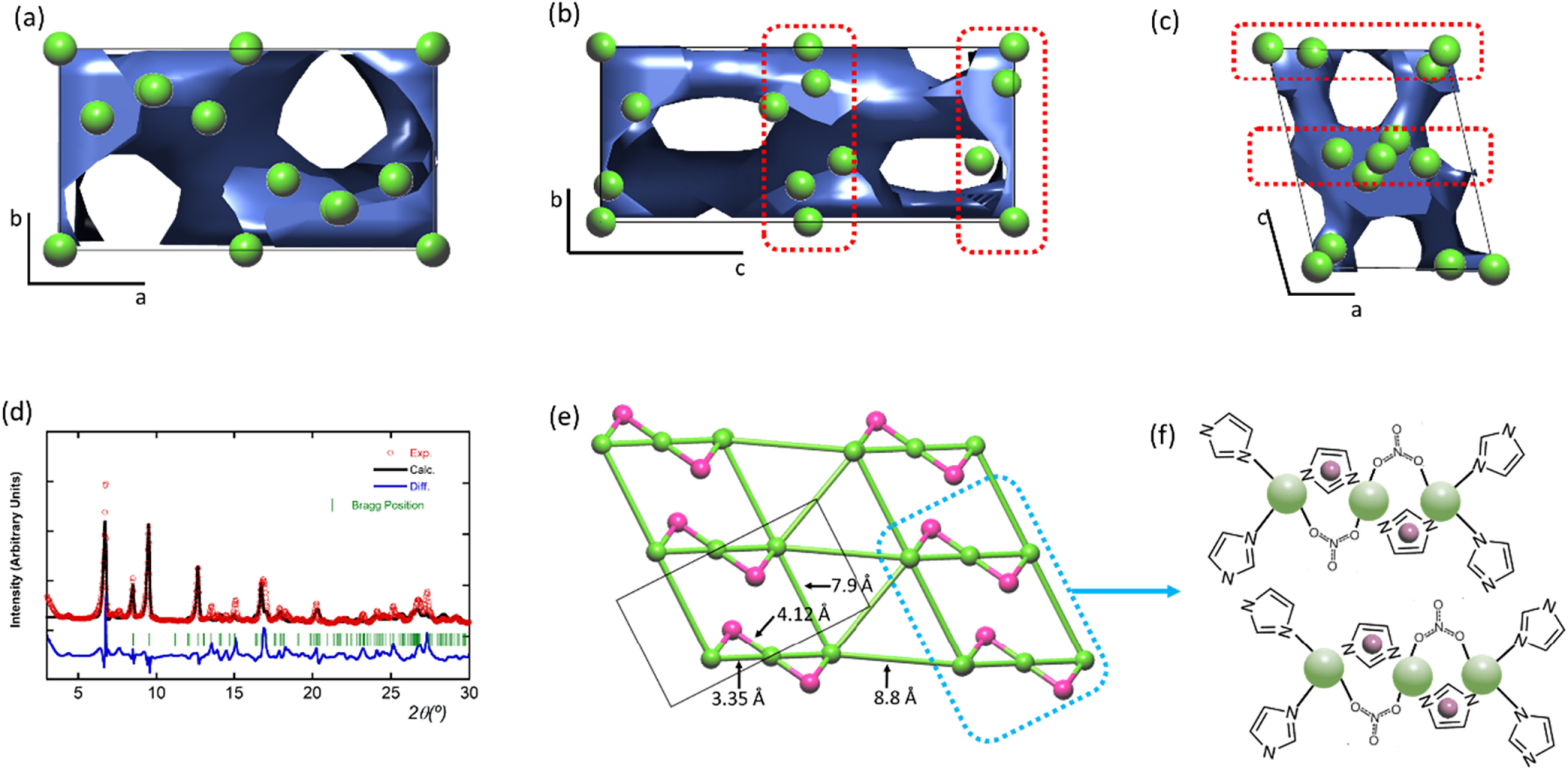
(a–c) Envelope electron density maps of ZIF-93_HP_3×(6.4) plotted in three different crystallographic directions. Green spheres stand for the Zn ions later located at high-electron rich regions (highlighted by the dotted-red line). (d) Final fitting of the Rietveld refinement taking into account a structural model built up from three independent Zn ions. (e) Simplification and connectivity of the structural model obtained from the Rietveld refinement. Green spheres correspond to Zn ions and pink ones to the average electron density positions arising from the mImca linker. (f) Tentative model built up from the estimated Zn–Zn distances.

From the envelop density map, the crystallographic positions of three electron-rich density points were assigned to three crystallographic independent Zn atoms, one of them located at a special position in the origin of the unit cell. In the next step, a Rietveld refinement of the data was performed with a structural model consisting of three Zn ions. At this stage, we attempted to introduce imidazolate and nitrate species into the structure using rigid block Rietveld refinement in an effort to locate them more precisely. However, the positioning of these components of the structure remains elusive, suggesting that current PXRD data is not accurate enough to model their presence within the crystal framework. Although the initial Rietveld analysis accounting just the Zn ions fits relatively well with the experimental data (Figure 7d), we need to be careful on the evaluation of the obtained atomic positions (Figure 7e). In the model, Zn–Zn distances ranging from 3.0 to 4.5 Å suggest that the Zn ions located in electron-density-rich planes are likely bridged by nitrate anions. If one of the Zn ions is interpreted as the averaged position of electron density corresponding to an imidazolate, we could tentatively propose the presence of Zn-imidazole bridges alongside the Zn–NO_3_–Zn linkages. This arrangement would provide a plausible explanation for the observed structural features (see Figure 7f). These short Zn–Zn connectivity maps could generate anionic discrete trimeric units with an average formula of Zn­(mImca)_2_(NO_3_)_0.66_. The Zn–Zn distances between the trimeric units, ranging from 7.5 to 8.0 Å, are too large to assume a direct connection through mImca linkers (see Figure 7e,f). Therefore, it is plausible that the electron density found between these discrete units is occupied by nitrate and ammonium ions belonging to the promotor employed in the hot-pressing-based synthesis, which would compensate for the anionic charge of the framework. This arrangement supports the proposed average formula in this study (explained below): Zn­(C_5_N_2_OH_5_)_2_·1.2­(NH_4_NO_3_)·(H_2_O), where the inclusion of these ions and water molecules stabilizes the overall structure. In fact, these electrostatic stabilized structures tend to be relatively highly soluble in water; a fact that could explain the transformation of ZIF-93_HP to ZIF-93, during the washing process. It is important to note that the proposed model is a preliminary assumption that would need further experimental confirmation. In fact, the above interpretation of the XRD-data does not represent a formal structural model, but this qualitative information suggests the formation of a nonextended coordination network that is consistent with the empirical formula and the other features determined by complementary experimental techniques. Finally, the current powder data has not enough information to completely support the refinement of such a complex and low symmetry structure.

### 3.5 ZIF-93_HP Washing and Its Characterization

To elucidate the processes occurring during the washing step with water, both supernatant and solid were analyzed. At a first presumption, the supernatant was examined to determine if a new laminar phase had been formed and an exfoliation was occurring when exposed to water. Figure 8a shows the XRD patterns for the sample before washing (ZIF-93_HP_3×(6.4)), the product deposited after washing with water and the solid recovered after centrifuging the supernatant. Both supernatant and deposited solid present similar crystal morphologies which correspond with that of ZIF-93 (rombododecahedral, as reported elsewhere^38,39^ ). These results discard the presumption of an exfoliation, revealing a transformation of ZIF-93_HP into the well-known ZIF-93 in the presence of polar solvents, as said above. Both PXRD data (Figure 8a) and SEM images (Figure 8b,c) confirmed the conversion showing similar morphologies to that of ZIF-93 for both samples, solid (Figure 8b) and supernatant (Figure 8c). The supernatant presented a more defined morphology that allows a better measurement of its particle size (150 ± 31 nm). The structural transformation of ZIF-93_HP into ZIF-93 is primarily justified by the changes observed in PXRD and SEM, which confirm the disappearance of the initial phase and the emergence of the ZIF-93 phase with its characteristic morphology. This transformation is further corroborated by gas adsorption analysis (later discussed), where a significant increase in N_2_ and CO_2_ uptake indicates the creation of a permanent porous network. While these collective results are robust for establishing the phase transformation, future characterization (such as in situ spectroscopy, NMR or synchrotron measurements) could provide complementary information about the structure of the new phase and its mechanism of transformation at the molecular level upon the removal of promotor NH_4_NO_3_ after washing with water.

**Figure 8:**
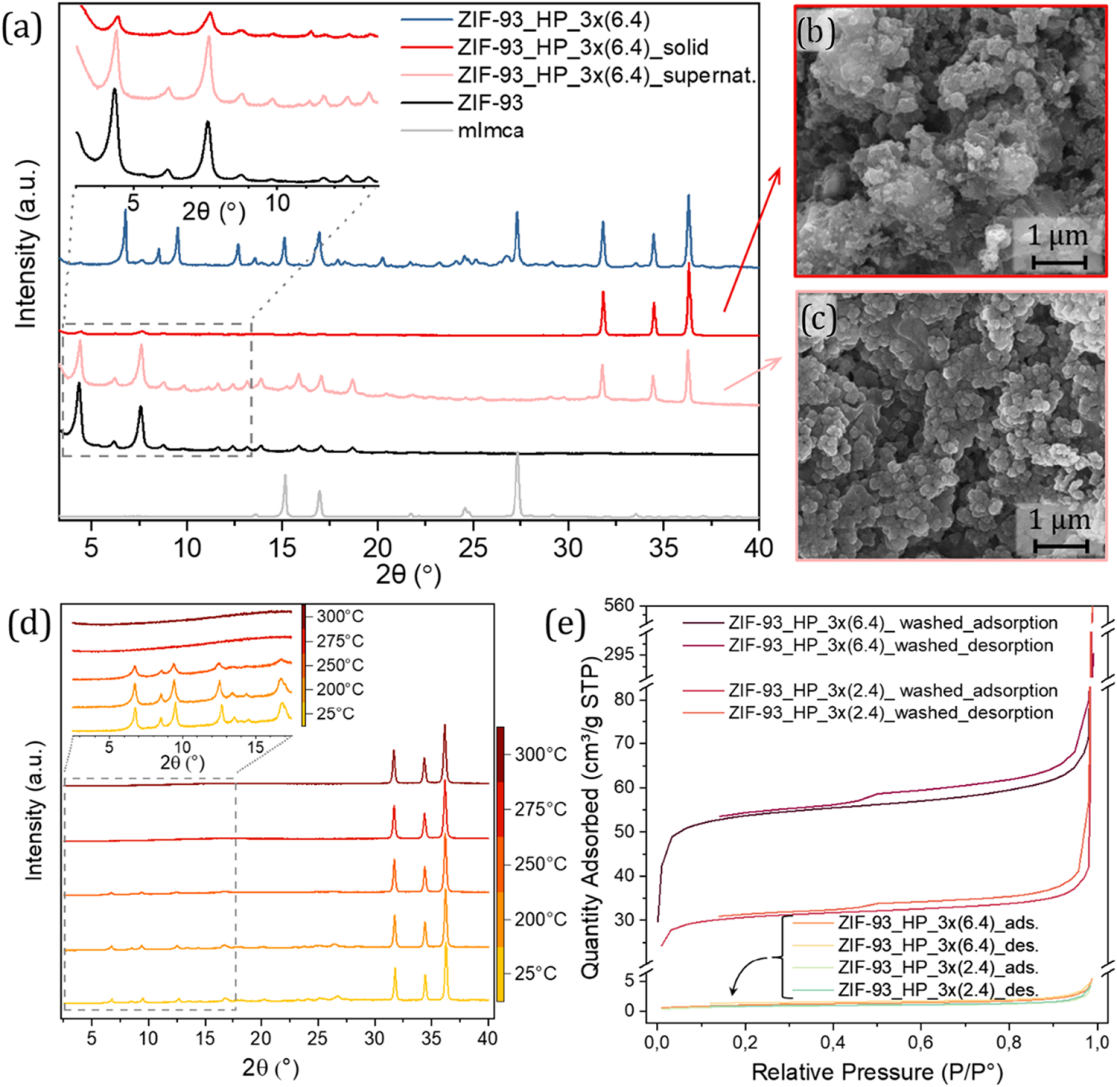
(a) PXRD patterns of sample ZIF-93_HP_3×(6.4) before washing (blue line), product deposited after washing with water (red line) and the solid after centrifuging the supernatant of the wash (pink line). On the right side, SEM image (at x50.000 magnifications) of (b) solid after washing with water and (c) supernatant centrifuged after washing with water. (d) X-ray thermodiffractometry from 25 to 300 °C of sample ZIF-93_HP_3×(6.4). (e) N_2_ adsorption–desorption isotherms of ZIF-93_HP_3×(2.4) and ZIF-93_HP_3×(6.4) before and after washing with water.

X-ray thermodiffractometry (XRTD) was employed to analyze the thermal evolution of the diffraction maxima ascribed to the ZIF-93_HP phase. ZIF-93_HP_3×(6.4) washed with OctOH and dried at 200 °C for 5 days was employed for this analysis. As shown in Figure 8d, the diffraction maxima ascribed to ZIF-93_HP phase remain constant up to 225 °C, temperature at which they start to loss intensity until they completely disappear above 275 °C. These results confirm that the structural stability of ZIF-93_HP is linked to the thermal removal of NH_4_NO_3_ from its structure. In contrast with the water-washing removal of NH_4_NO_3_, its thermal release induces its complete amorphization instead of its recrystallization into ZIF-93 (as happened when eliminating NH_4_NO_3_ with water). There is a slight mismatch between the degradation temperatures observed by TGA and XRTD, but it is reasonable considering the difference on the heating ramp and prolonged exposition of the sample to each temperature of measurement in XRTD (keeping each temperature step during 135 min, i.e., prolonging the exposure to high temperature as compared to the TGA conditions).

N_2_ adsorption also confirmed the transformation from a nonporous ZIF-93_HP to the typical RHO open structure of ZIF-93. As expected, the conversion of ZIF-93_HP to ZIF-93 leads to a considerable increase in the BET SSA (see Table 2). ZIF-93_HP_3×(2.4) and ZIF-93_HP_3×(6.4) were analyzed before and after washing with water. Before washing, they showed BET SSA values of 3.1 ± 0.0 and 4.0 ± 0.0 m^2^/g, respectively, while after washing their surface area increased to 103.7 ± 1.7 and 181.3 ± 3.0 m^2^/g, respectively. N_2_ adsorption isotherms employed to calculate the BET SSA values are shown in Figure 8e. These results suggest that ZIF-93_HP either is a dense phase (not accessible to the N_2_ molecule) or its porosity is filled with water and NH_4_NO_3_. After washing ZIF-93_HP with water, despite the clear transformation to ZIF-93 indicated by PXRD (Figure 8a), the BET SSA is considerably lower than the reported for solvothermal ZIF-93 (in the ca. 600–900 m^2^/g range^21,38^ ). This may be related to the presence of ZnO in the inner part of the particles due to incomplete reaction of ZnO to ZIF-93_HP, evidenced in PXRD peaks of ZnO at 31.8, 34.5 and 36.3° (see Figure 3). This residual, nonporous ZnO acts as a mass diluent, significantly decreasing the apparent surface area per total mass of the sample. While residual promotor occlusion could also play a minor role, the presence of crystalline ZnO is the dominant factor when considering the adsorption properties of the material.

**Table 2:** BET SSA Values for Samples ZIF-93_HP_3×(2.4) and ZIF-93_HP_3×(6.4) before and after Washing with Water

	BET SSA (m^2^/g)	
sample	before washing	after washing
ZIF-93_HP_3×(2.4)	3.1 ± 0.0	103.7 ± 1.7
ZIF-93_HP_3×(6.4)	4.0 ± 0.0	181.3 ± 3.0

To further probe the microporosity and eliminate the possibility of permanent pore blockage, CO_2_ adsorption was measured at 273 K (see Figure S7). The results showed a measurable uptake increasing from 0.44 mmol/g for ZIF-93_HP to 0.54 mmol/g for the washed phase. This confirms the presence of accessible micropores in the transformed ZIF-93 framework. The moderate uptake values align with the dilution effect caused by the presence of nonporous, unreacted ZnO, which reduces the apparent gas adsorption capacity when normalized to the total mass of the sample.

The different weight loss steps observed in the TGA curve from sample ZIF-93_HP_3×(6.4) washed with OctOH and dried at 200 °C for 5 days (Figure S6c) were used to calculate the molar ratio of each component in the sample. Based on the analysis, the 18.7% of the weight is ascribed to structural NO_3_^–^, 42.0% to structural ligand, and the 39.4% to ZnO, from which 15.7% belongs to ZnO coming from the calcination of the ZIF-93_HP sample (calculated from the amount of ligand and the 2:1 ligand:Zn molar ratio of the ZIF) and 23.7% to unreacted ZnO. All in all, Zn­(C_9.9_N_6.4_O_6.6_H_14.8_) can be proposed as the core formula for ZIF-93_HP. Considering water and NH_4_NO_3_ as structural parts of ZIF-93_HP, the following empirical formula is proposed: Zn­(C_5_N_2_OH_5_)_2_·1.2­(NH_4_NO_3_)·(H_2_O).

The content of C, N and H calculated from TGA was compared with the one obtained from elemental analysis. First, the C, N and H content (3.27, 1.52 and 0.33%, respectively) of the unreacted ligand (6.0%) present in the sample washed with toluene (calculated from the weight loss around 200 °C in the TGA shown Figure S6a) was subtracted from the elemental analysis results. After this correction, the experimental and calculated values obtained from the proposed formula closely agree. These values are collected in Table 3.

**Table 3:** Contents of C, N, H from Elemental Analysis of Sample ZIF-93_HP_3×(6.4) after Washing with Toluene and from the Proposed Empirical Formula (Zn­(C_5_N_2_OH_5_)_2_·1.2­(NH_4_NO_3_)·(H_2_O))

	C (%)	N (%)	H (%)
elemental analysis	35.3	22.7	3.6
elemental analysis corrected (subtracting 6.0% of unreacted ligand)	32.0	22.2	3.3
empirical formula	30.1	22.6	4.3

All the experimental clues point that the transformation of ZIF-93_HP into ZIF-93 is related to the removal of NH_4_NO_3_. This salt, besides promoting the deprotonation and reaction of precursors (shown through eqs 3–5), could act as structure directing agent forming an integral part of the framework of ZIF-93_HP, as suggested by the preliminary structural analysis. The irreversible transformation of ZIF-93_HP into the three-dimensional framework of ZIF-93 upon washing with water is consistent with a topotactic solid-state reorganization mechanism. Analogous to the transformation of ZIF-L to ZIF-8,^40,41^ the removal of the NH_4_NO_3_ likely creates structural instability, promoting a rearrangement of the framework into its more thermodynamically stable phase ZIF-93.

### 3.6 Protonic Conductivity of ZIF-93_HP

The proton conductivity of the sample was evaluated by complex electrochemical impedance spectroscopy (EIS). Figure 9 shows typical Nyquist diagrams obtained at different temperatures with a relative humidity of ca. 97%. The recorded spectra show the characteristic inclined line associated with proton diffusion processes.

**Figure 9:**
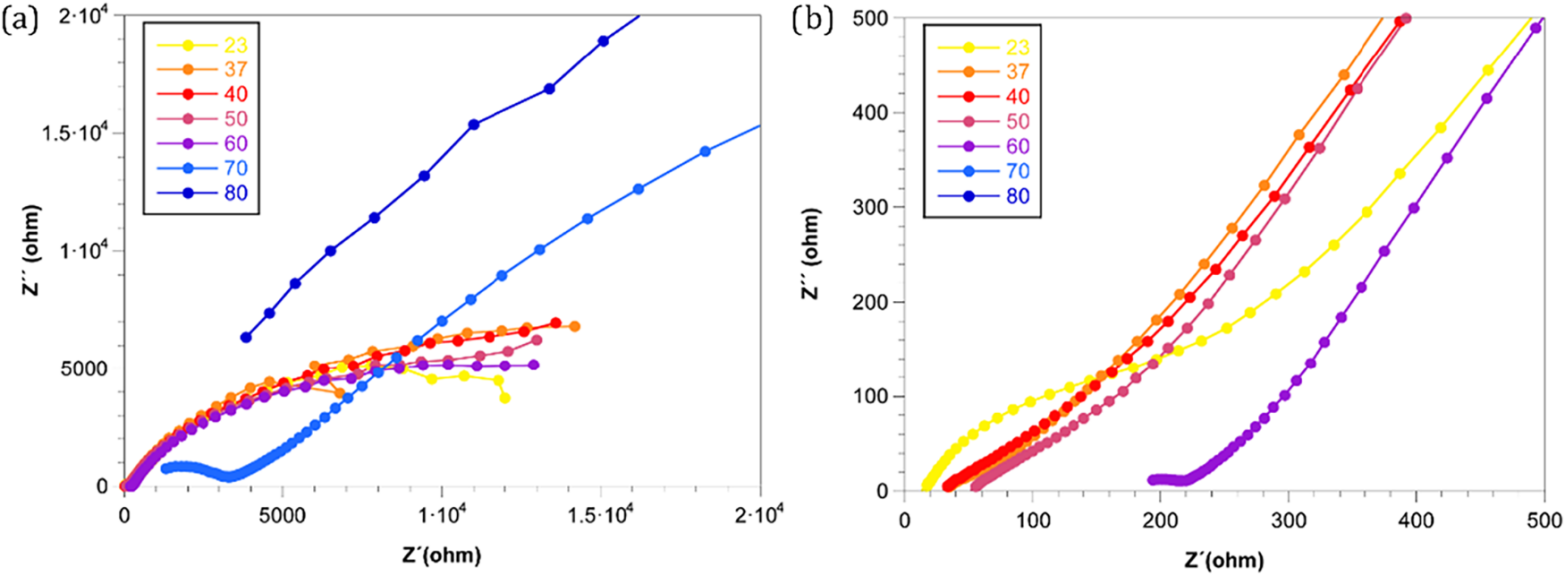
(a) Nyquist plot of the ZIF-93_HP sample measured in the temperature range of 23–80 °C at ca. 97% RH. (b) Highlight of the Nyquist plot data for these samples measured showing higher proton conductivities.

The resistance at each temperature was estimated from the high frequency end of the straight line and the ionic conductivity was calculated through eq 2, obtaining the values shown in Figure 10. The absence of mixed valences that could introduce charge carriers into the compound and the difficulty in developing long-range charge transport pathways allows us to rule out an efficient contribution from electrical conductivity. At ambient humidity (ca. 50% RH) and R.T. the as-synthesized ZIF-93_HP has a conductivity of 2.19 × 10^–5^ S/cm at 23 °C. The presence of humidity allows a higher adsorption of water molecules, also providing a greater mobility of protons through the material and influencing the proton conductivity. Thus, the ionic conductivity at 23 °C in the presence of ca. 97% RH increases to 3.76 × 10^–3^ S/cm, which implies an improvement of 2 orders of magnitude and which is considerably high for MOF type materials. However, those containing carboxylic groups and Cr/Mn metallic centers can reach conductivities in the range of 10^–2^–10^–3^ S/cm at 25 °C and 98% RH, but decreasing orders of magnitude for just a slight decrease in RH or above 50 °C.^42,43^

**Figure 10:**
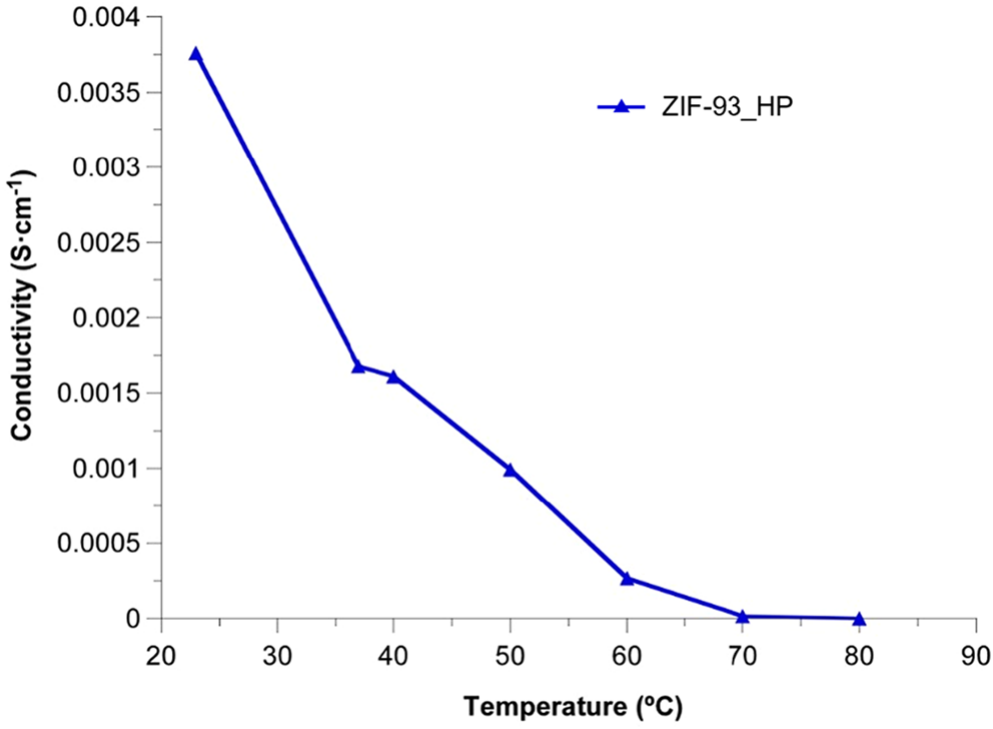
Proton conductivity as a function of temperature at ca. 97% RH for ZIF-93_HP.

As the temperature rose, a decrease in conductivity was observed (Figure 10). On one hand, the mobility of protons increases with temperature and, therefore, this is expected to increase conductivity. On the other hand, the number of protons decreases with temperature as a result of the loss of water molecules, and this is expected to produce a decrease of conductivity. It is worth mentioning that, based on the conductivity values obtained for ZIF-93_HP (σ > 10^–4^ S/cm), this material can be considered as a superionic conductor or fast ionic conductor.^44^ The promising ionic-conductive properties of ZIF-93_HP stand in contrast to its limited chemical stability, which restricts its direct application in aqueous-based fuel cell systems, and most likely in the long term in proton exchange membrane fuel cells (PEMFCs) operated under humid gas streams. Still, studying the proton conduction of materials such as ZIF-93-HP, which host salt-like species like ammonium nitrate in their framework, offers fundamental insights that could guide the development of more robust proton conductors with similar features.

Through the extrapolation of our prior work about the synthesis of ZIF-8 and ZIF-L with hydraulic press and high temperature,^28^ notable discoveries have to be highlighted. Apart from the successful synthesis of ZIF-93 (after the subsequent washing with water), our efforts revealed a novel ZIF related phase (ZIF-93_HP). This unique phase probably has not been reported previously due to the necessity of solvent-free conditions for its crystallization. The formation of the ZIF-93_HP phase under high-pressure, solvent-free conditions may be indicative of a kinetic trapping mechanism. The combination of ex-situ analysis and in situ temperature-dependent XRD offers evidence for this pathway. The observed formation of ZIF-93_HP within a specific and moderate temperature range, as well as its irreversible transformation to the thermodynamic product ZIF-93 upon removal of the NH_4_NO_3_ promotor, are characteristic indications of a metastable intermediate. This evidence supports the conclusion that the ZIF-93_HP framework is stabilized kinetically by the unique synthesis environment and the presence of NH_4_NO_3_.

The proposed structural model for ZIF-93_HP represents an idealization. The potential presence of framework defects, such as missing linkers or localized amorphous regions, could significantly influence the functional properties of the material. Such imperfections may enhance proton conductivity by facilitating water uptake but could also alter continuous proton-transfer pathways. They likely contribute to the material thermal stability profile by introducing points of weakness in the framework. The influence of these factors highlights the complex structure–property relationship in metastable ZIF phases synthesized under nonequilibrium conditions.

All in all, this discovery emphasizes the value of innovative synthetic techniques and presents new opportunities for MOF research. It is reasonable to anticipate that similar synthesis approaches applied to other ZIFs may lead to additional novel phases under these singular conditions, even if some challenges remain about the structural characterization of the sample and the own scalability of the HP synthesis procedure.

## 4 Conclusion

The high-pressure and temperature synthesis method employed in this study offers a promising route for the solventless synthesis of MOFs beyond conventional thermodynamic equilibrium. Here, we specifically focused on the green production of nanoporous ZIF-93 through the crystallization of a previously unreported intermediate phase, ZIF-93_HP. Conducted at 110 °C and 150 MPa, this approach yielded nanosized, fiber-like ZIF-93_HP crystals (∼ 50 nm thick). The addition of NH_4_NO_3_ as a promotor significantly improved the synthesis yield and further investigation into the influence of reaction steps and washing with different solvents provided valuable insights about the transformation of ZIF-93_HP into ZIF-93. The identification of the mentioned novel phase highlights the potential for innovation in MOF synthesis and suggests the possibility of finding new MOF phases crystallized under unconventional and solventless conditions.

Even if further work is needed to fully understand the structural and chemical properties of ZIF-93_HP, we have been able to propose its empirical formula Zn­(C_5_N_2_OH_5_)_2_·1.2­(NH_4_NO_3_)·(H_2_O) by the combination of thermogravimetry and elemental analysis. Further, ED data allowed the determination of the *P*2/*n* space group (with unit cell constants *a* = 14.82 Å, *b* = 7.69 Å, *c* = 18.45 Å, α = γ = 90.000° and β = 100.889°), while the subsequent Rietveld refinement of the PXRD produced the first insights into the structure model of ZIF-93_HP. The structural arrangement supports proton conductivity under humid conditions at room temperature. Although an increase in temperature would typically enhance proton conductivity, in this case, it is negatively offset by a reduction in the ionic mobility due to water loss from the sample. This research contributes to the understanding of MOF synthesis and opens the room to the crystallization of novel phases out from the conventional synthesis paths.

## Supplementary Material



## Notes

Photographs of the experimental setup (Figure S1), a comparative table of different synthesis methods (Table S1) and additional characterization: TGA (Figures S2b, S3b and S6), XRD (Figures S2a, S3a, S4 and S5) and CO_2_ adsorption isotherms (Figure S7) (PDF)
