# Supplementary material for: High Pressure-Based Synthesis of Nanoporous Metal–Organic Framework ZIF-93 Giving Rise to a Phase for Proton Conduction
Source: ACS Appl Nano Mater. 2025 Oct 18;8(43):20713–25. doi: 10.1021/acsanm.5c03130 (PMC12584345; doi:10.1021/acsanm.5c03130)
Supplement: Supplementary file 1 [file an5c03130_si_001.pdf]

SUPPORTING INFORMATION

High Pressure-Based Synthesis of Nanoporous Metal-Organic Framework ZIF-93 Giving Rise to a Phase for Proton Conduction

Marta Pérez-Miana[1,2], Roberto Fernández de Luis [3], Arkaitz Fidalgo-Marijuan[3,4], Junyan Li[5,6], Álvaro Mayoral[1], Joaquín Coronas[1,2,*]

[1]Instituto de Nanociencia y Materiales de Aragón (INMA), CSIC-Universidad de Zaragoza, Zaragoza, 50018, Spain

[2]Chemical and Environmental Engineering Department, Universidad de Zaragoza, Zaragoza, 50018, Spain

[3]Basque Center for Materials, Applications and Nanostructures (BCMaterials), UPV/EHU, Leioa, 48940, Spain

[4]Department of Organic and Inorganic Chemistry, University of the Basque Country UPV/EHU, 48940, Leioa, Spain

[5]Centre for High-resolution Electron Microscopy (ChEM), School of Physical Science and Technology, ShanghaiTech University, Shanghai, 201210, China

[6]State Key Laboratory of Inorganic Synthesis and Preparative Chemistry, College of Chemistry, Jilin University Changchun 130012, China

*Corresponding author: Joaquín Coronas (coronas@unizar.es)

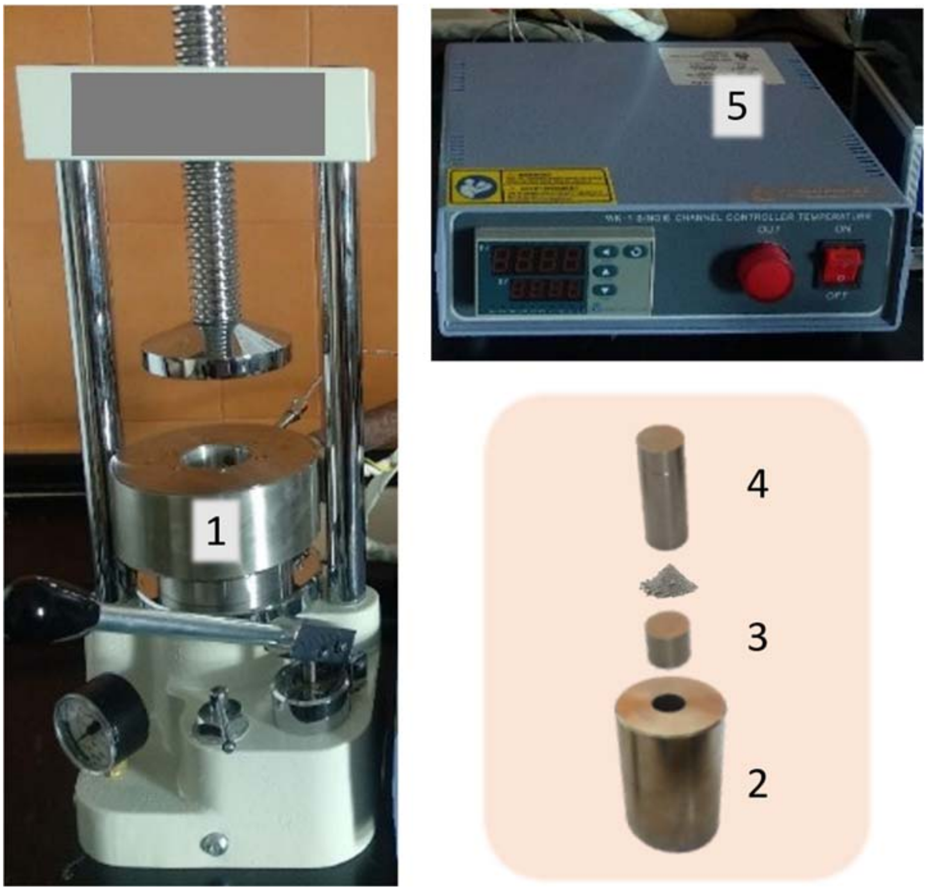

Figure S1. Hydraulic press setup: 1 – Heating cylinder, 2 – Steel sleeve (die) or cylinder, 3 – Pushing rod (downer one/base), 4 – Pushing rod (upper one/piston) and 5 – heating controller.

Table S1: Comparison of synthesis parameters for 3 different syntheses of ZIF-93 and the novel ZIF-93_HP.

	**ZIF-93** (Solvothermal) [13]	**ZIF-93** (Hydrothermal) [21]	**ZIF-93** (Hydrothermal) [22]	**This Work: ZIF-93_HP**
**Synthesis Method**	Solvothermal	Hydrothermal	Solvothermal	**Solvent-Free, HP**
**Solvent (reaction step)**	DMF	Water	Water	**None**
**Temperature**	85 °C	Room temperature	Room temperature	**110 °C**
**Time**	12 h	5 minutes	18 h	**10/30 minutes (x3)**
**Pressure**	Ambient	Ambient	Ambient	**150 MPa**
**Ligand:Metal Ratio**	1 : 4	8 : 1	2 : 1	**2 : 1**
**Additive**	-	-	NH_4OH	**NH_4NO_3 (Promotor/Template)**
**Key Advantage**	Original synthesis	Eco-friendly solvent, Nanocrystals	Eco-friendly solvent and not ligand excess	**Novel phase, Solvent-free, Stoichiometric, Proton conductivity**
**BET SSA (m^2/g)**	~ 864	~ 1309	~ 604	**~180**
**Proton Conductivity (S/cm)**	Not typically reported	Not reported	Not reported	**3.76×10^{-3}**

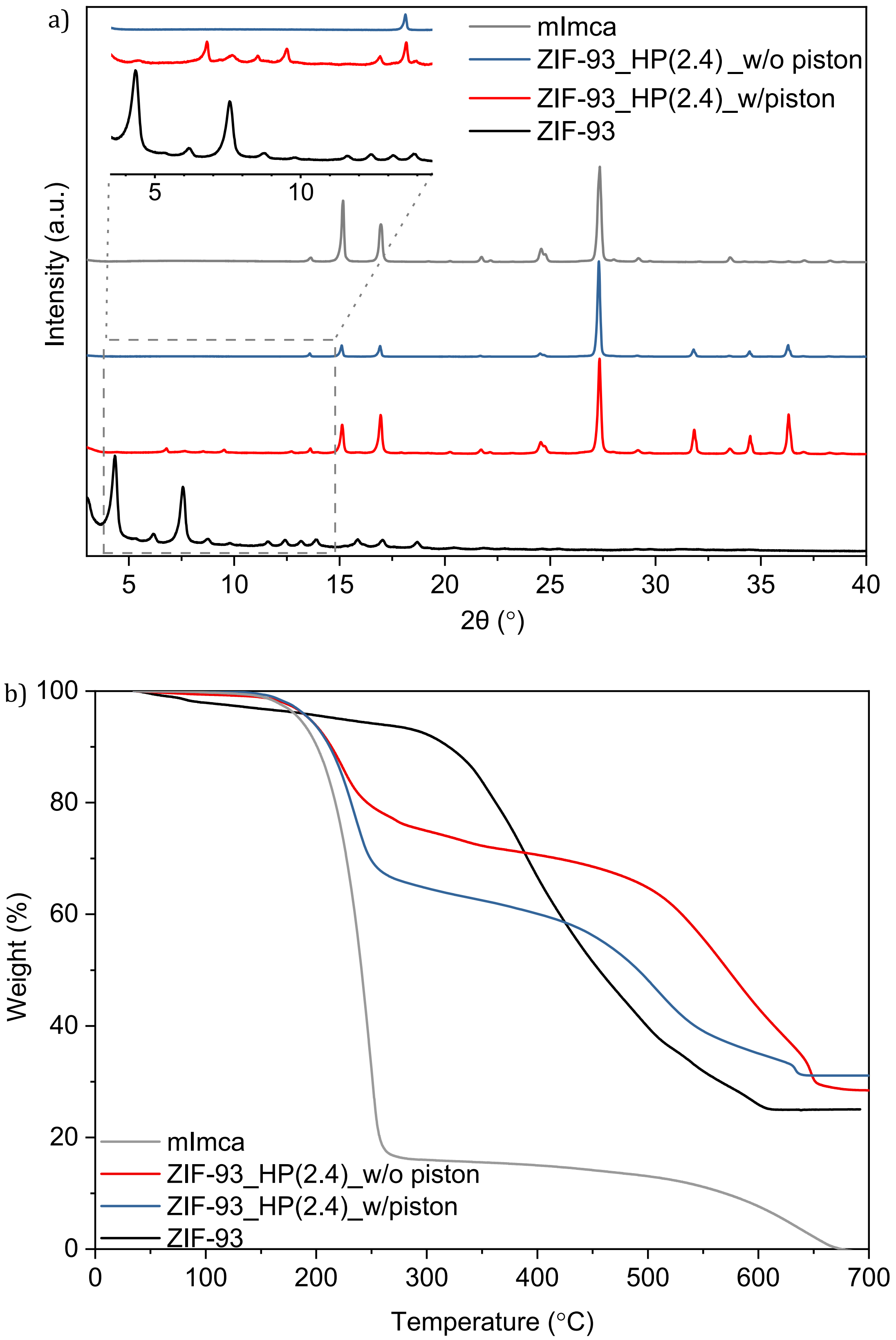

Figure S2. XRD patterns (a) and TG curves (b) of samples without pressure, one of them compacted using the piston (blue line) and the other without using it (red line). ZIF-93 and mImca have been included for comparison.

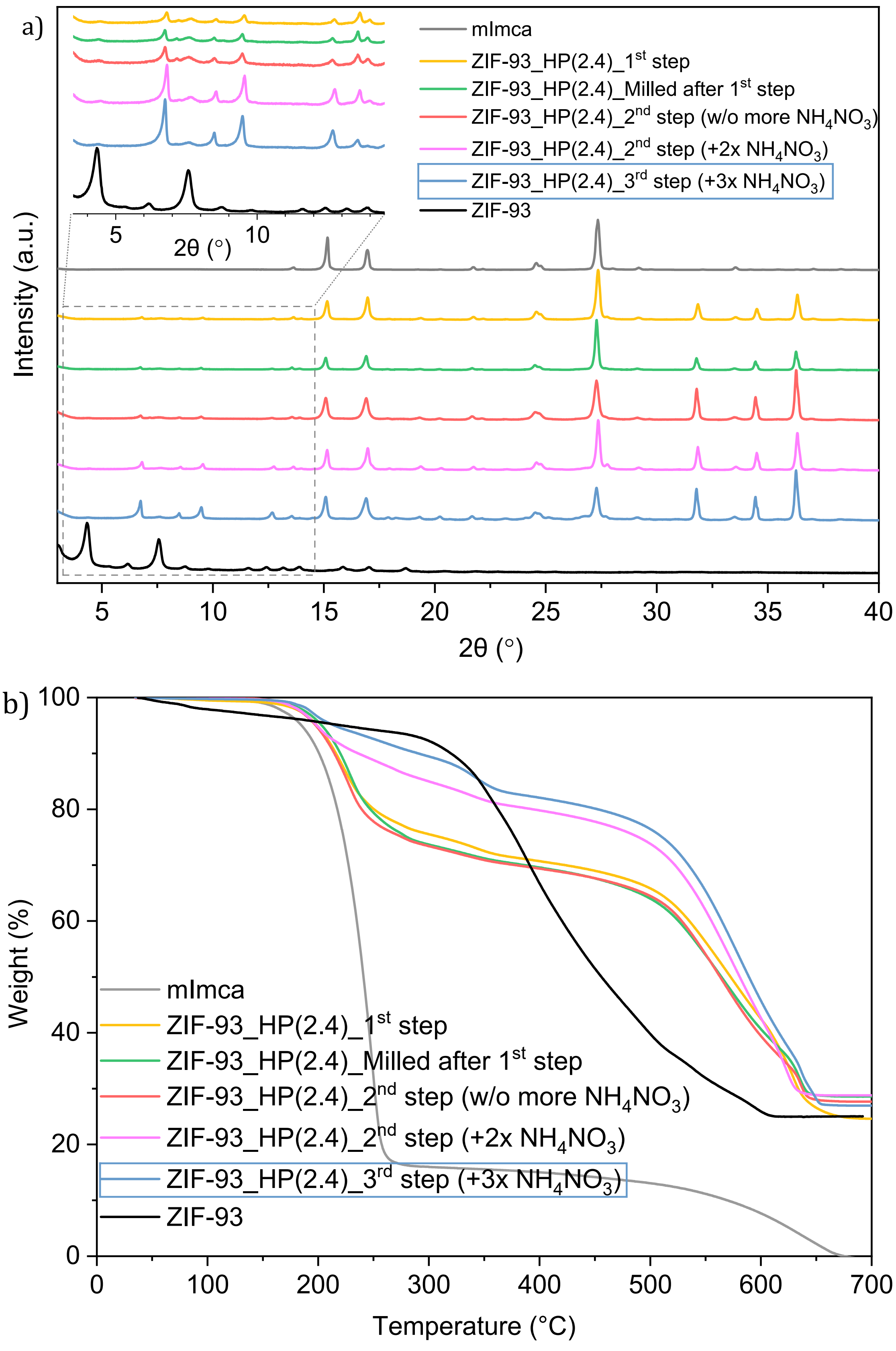

Figure S3. XRD patterns (a) and TGA (b) of sample ZIF-93_HP(2.4) and different reaction steps.

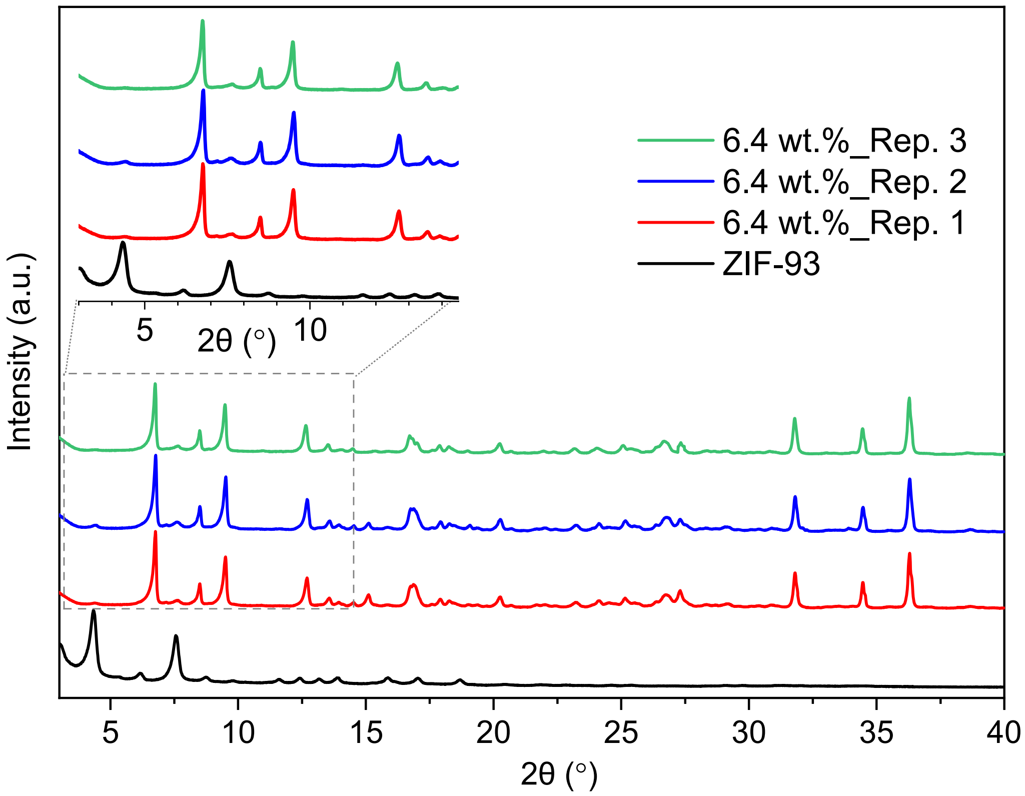

Figure S4. PXRD patterns of three independently synthesized batches of ZIF-93_HP using the 3x6.4 wt% promoter condition, demonstrating the reproducibility of the phase formation.

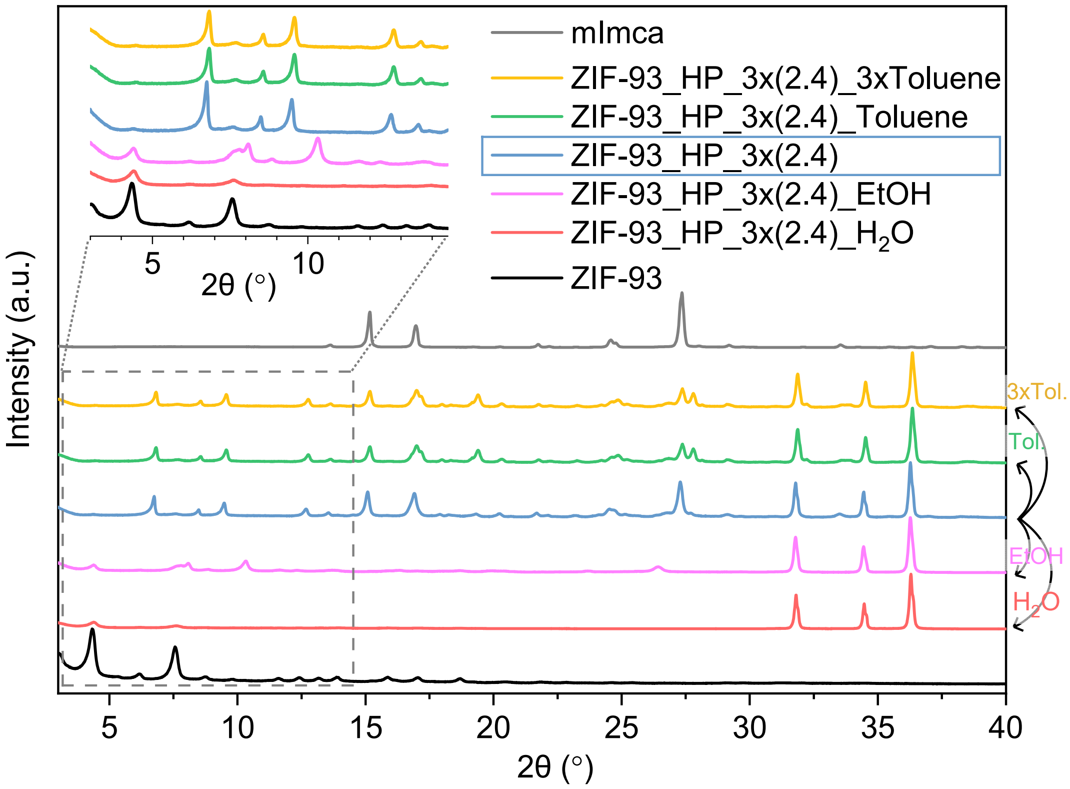

Figure S5. XRD patterns of sample ZIF-93_HP_3x(2.4) after washing with different solvents: water (red), ethanol (pink), toluene (green) and 3 washes of toluene (yellow).

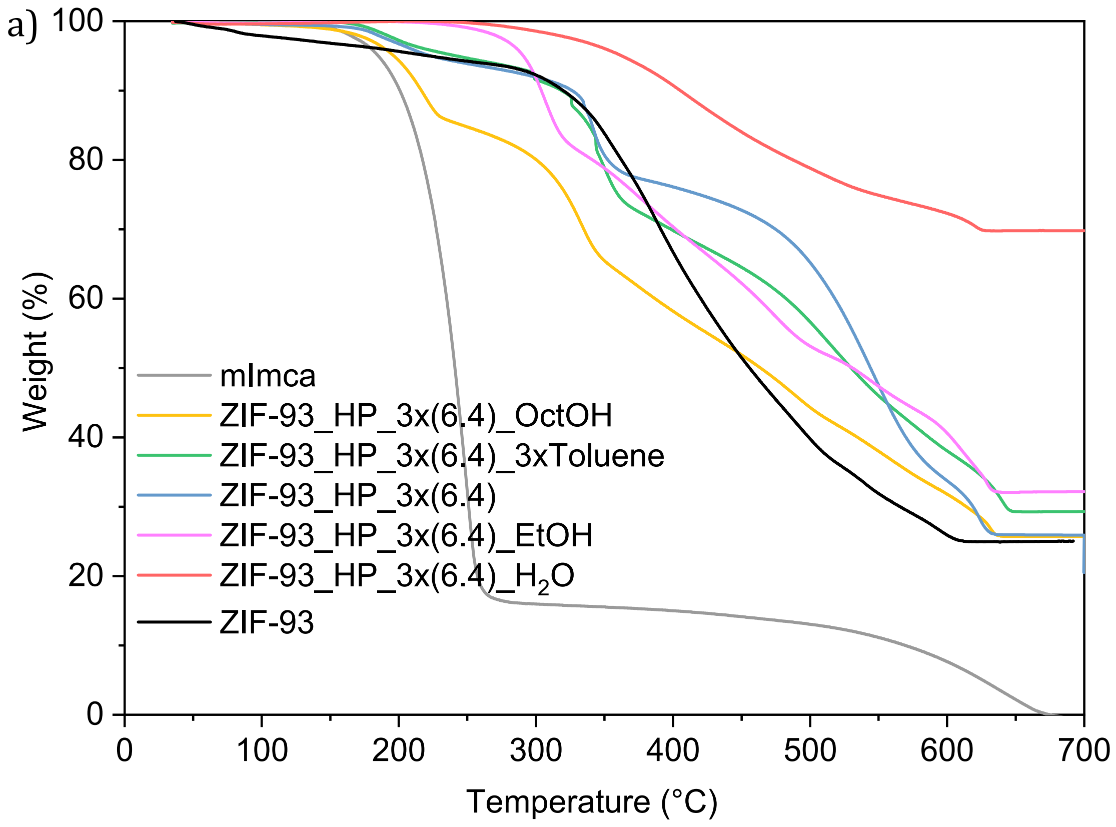

a)
100
80
60
40
20
0
Weight (%)
0
100
200
300
400
500
600
700
Temperature (°C)
mImca
ZIF-93_HP_3x(6.4)_OctOH
ZIF-93_HP_3x(6.4)_3xToluene
ZIF-93_HP_3x(6.4)
ZIF-93_HP_3x(6.4)_EtOH
ZIF-93_HP_3x(6.4)_H_2O
ZIF-93

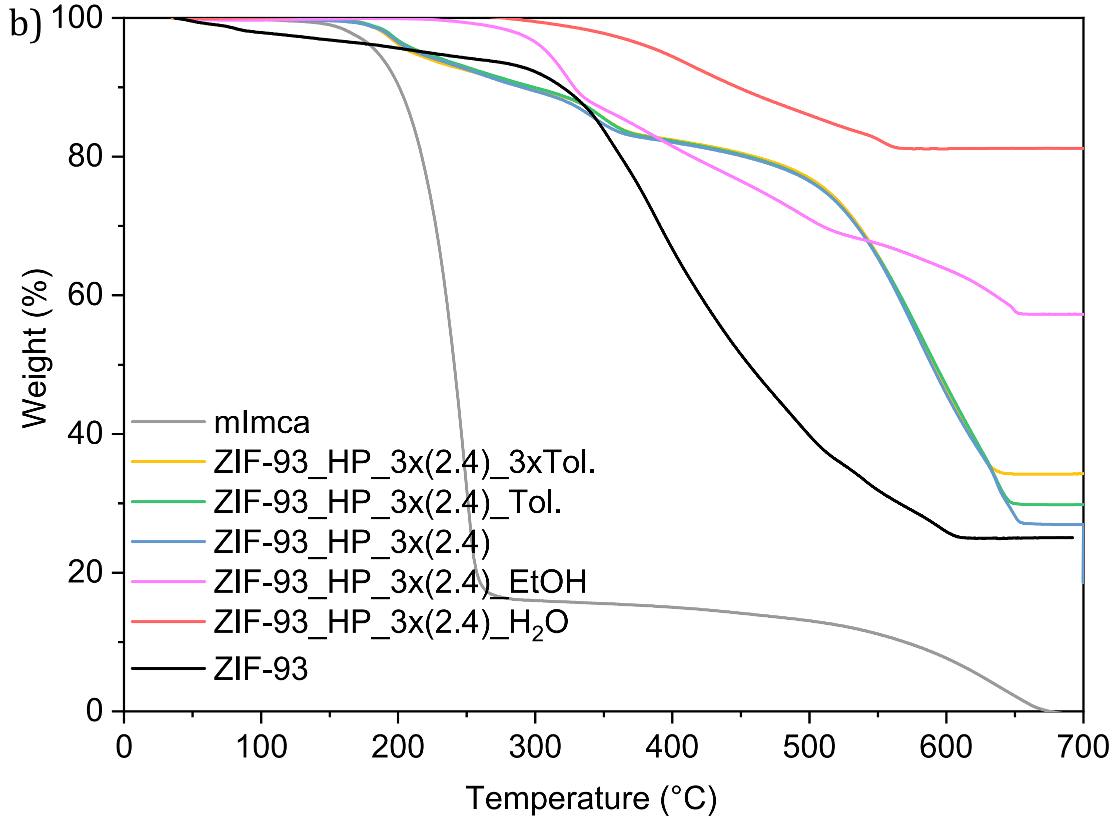

b)
100
80
60
40
20
0
Weight (%)
0
100
200
300
400
500
600
700
Temperature (°C)
mImca
ZIF-93_HP_3x(2.4)_3xTol.
ZIF-93_HP_3x(2.4)_Tol.
ZIF-93_HP_3x(2.4)
ZIF-93_HP_3x(2.4)_EtOH
ZIF-93_HP_3x(2.4)_H_2O
ZIF-93

c)
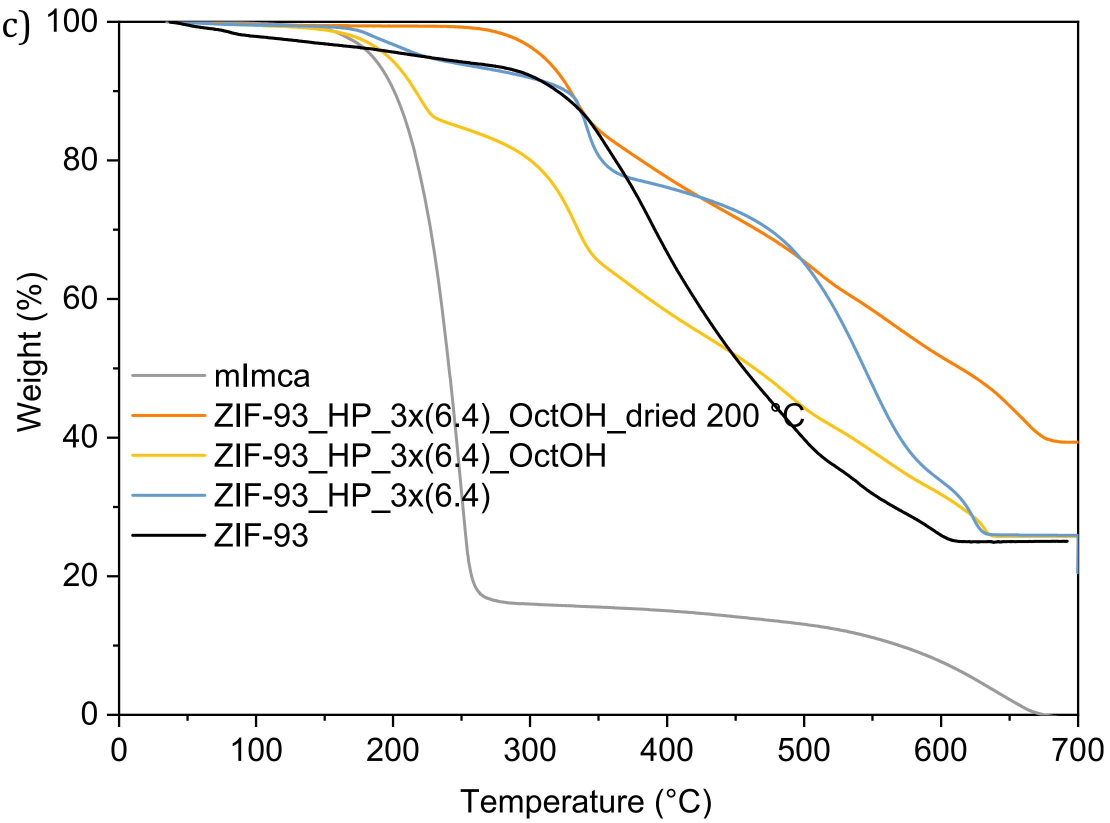

Figure S6. TGA curves of sample a) ZIF-93_HP_3x(6.4), b) ZIF-93_HP_3x(2.4) after washing with different solvents and c) sample ZIF-93_HP_3x(6.4) before washing, after washing with OctOH and its subsequent drying at 200 °C for 5 days.

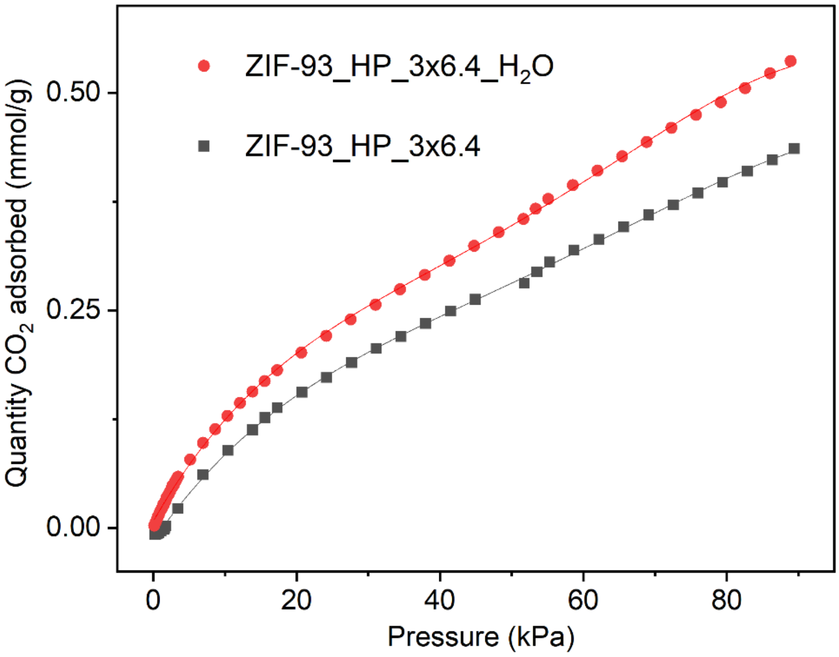

Figure S7. CO_2 adsorption isotherms of ZIF-93_HP_3x6.4 before and after washing with H_2O.
